# Clinical Characteristics, Risk Score Distribution, and Hospitalization Status in Emergency Department Patients with Acute Chest Pain: A Single-Center Retrospective Four-Year Study

**DOI:** 10.3390/clinpract16060103

**Published:** 2026-05-29

**Authors:** Gabriela-Florentina Țapoș, Dan Iliescu, Mihaela Cristina Negru, Florin Borcan, Silvia Luca, Simina Crișan, Constantin Tudor Luca

**Affiliations:** 1 Doctoral School, “Victor Babes” University of Medicine and Pharmacy, Eftimie Murgu Square 2, 300041 Timisoara, Romania; gabriela.tapos@umft.ro; 2 Arad County Clinical Emergency Hospital, 310037 Arad, Romania; 3 Department of Surgery I, Clinic of Surgical Semiotics & Thoracic Surgery, “Victor Babes” University of Medicine and Pharmacy, Eftimie Murgu Square 2, 300041 Timisoara, Romania; dan.iliescu@umft.ro; 4 Center for Hepato-Biliary and Pancreatic Surgery, “Victor Babes” University of Medicine and Pharmacy, Eftimie Murgu Square 2, 300041 Timisoara, Romania; 5 Department of Ear, Nose and Throat, “Victor Babes” University of Medicine and Pharmacy, Eftimie Murgu Square 2, 300041 Timișoara, Romania; 6 Department I (Analytical Chemistry), Faculty of Pharmacy, “Victor Babes” University of Medicine and Pharmacy, Eftimie Murgu Square 2, 300041 Timisoara, Romania; fborcan@umft.ro; 7 Cardiology Department, “Victor Babes” University of Medicine and Pharmacy, 300041 Timisoara, Romania; silvia.luca@umft.ro (S.L.); simina.crisan@umft.ro (S.C.); constantin.luca@umft.ro (C.T.L.); 8 Institute of Cardiovascular Diseases Timisoara, 300310 Timisoara, Romania; 9 Research Center of the Institute of Cardiovascular Diseases Timisoara, 300310 Timisoara, Romania

**Keywords:** acute chest pain, cardiovascular risk, risk stratification, emergency department

## Abstract

Background/Objectives: Cardiovascular diseases are a major cause of morbidity and mortality worldwide. Acute chest pain is a frequent reason for emergency department presentation and requires structured evaluation to identify life-threatening conditions. This study evaluated clinical characteristics, cardiovascular risk profile, risk stratification patterns, and hospitalization status in adults with acute chest pain. Methods: We conducted a retrospective study using registry data from Arad County Clinical Emergency Hospital between January 2021 and December 2024. Adult patients with documented acute chest pain were included according to predefined criteria. Demographics, comorbidities, clinical presentation, troponin values, hospitalization status, and HEART, and EDACS categories were extracted when available. The Marburg Heart Score was also assessed as an exploratory complementary score. Statistical analysis used descriptive statistics, contingency tables, and chi-square testing, with available-case analysis. Results: Overall, 2070 patients were included. Most patients were aged 35–54 or 55–69 years. Hypertension and diabetes mellitus were the most common comorbidities, and pressure-like chest pain predominated. In unadjusted analyses, HEART and EDACS categories were significantly associated with hospitalization status across all study years. Score categories were significantly associated with hospitalization status across all study years. Age was consistently associated with cardiovascular comorbidity burden and higher-risk score categories. Conclusions: Structured risk stratification scores were associated with hospitalization status, while age was associated with cardiovascular risk burden.

## 1. Introduction

Cardiovascular diseases represent a major cause of morbidity and mortality worldwide, accounting for nearly one-third of all deaths and placing a substantial socioeconomic burden on healthcare systems [1].

Chest pain is very common in the general population, with approximately 25% of individuals facing it at least once in their lifetime. When patients present to the emergency department, the first step is to identify life-threatening conditions such as acute coronary syndromes, aortic dissection, aortic aneurysm rupture, and tension pneumothorax [2,3]. A systematic approach is necessary for the evaluation of chest pain. Based on the patient’s medical history and physical examination, additional tests may be performed, such as an electrocardiogram (ECG), a chest X-ray, or other imaging studies. Initial treatment may include oxygen administration or use of medications such as aspirin, nitroglycerin, anticoagulants, or beta-blockers. Depending on the clinical outcome, patients may subsequently require rapid transfer to a specialized medical facility, and in some cases, monitoring and treatment in the intensive care unit may be necessary [4,5].

However, despite these diagnostic strategies, determining the exact cause of chest pain remains challenging in clinical practice. At the same time, unnecessary tests and hospitalizations contribute to rising healthcare costs, highlighting the need to optimize diagnostic and treatment strategies [6]. Effective communication of the medical team and the use of standardized protocols for evaluating chest pain can help reduce unnecessary hospital admissions and improve diagnostic accuracy and patient outcomes [7]. In the emergency department, the focus is on identifying patients at highest risk, as well as those with non-urgent conditions who can be discharged after a minimum number of investigations or procedures. Non-essential investigations can expose patients to treatment-related risks and lead to increased medical costs. Therefore, risk scores were developed to help in the assessment of patients with chest pain and to identify those at higher risk of complications [8]. Although there are several risk assessment instruments for evaluating acute chest pain in the emergency department, evidence regarding their use in daily clinical practice and their association with hospitalization outcomes is limited. Assessing the HEART, EDACS, and Marburg scores within the same patient population could provide additional insights into risk assessment in the emergency department and the clinical decision-making process.

In this context, the aim of the present study was to evaluate the clinical characteristics, cardiovascular risk profile, and risk stratification patterns of patients presenting with acute chest pain in a tertiary care emergency department, and to explore the associations between demographic factors, comorbidities, risk scores (HEART, EDACS, and Marburg), and hospitalization status.

## 2. Materials and Methods

### 2.1. Study Design

We conducted an observational, retrospective study using routinely collected hospital registry data from Arad County Clinical Emergency Hospital (Arad, Western Romania). The study period (January 2021–December 2024) refers to the timeframe analyzed in the retrospective study, specifically the period from which the data for the patients included in the analysis were drawn. Data collection/extraction from the hospital archive began on 8 November 2024, after obtaining approval from the Hospital Ethics Committee. Data were extracted from institutional registries and patient medical records documented during the index episode of care.

### 2.2. Study Population

All consecutive patients presenting to the emergency department with chest pain and recorded in the hospital registries during the study period were screened for eligibility. To this study, acute chest pain was defined as chest discomfort, pressure-like pain, stabbing pain, burning pain, or other thoracic pain symptoms documented as the main or clinically relevant presenting complaint during the index emergency department encounter. Inclusion criteria were: (i) chest pain documented in the registry or medical record; (ii) age ≥ 18 years old; and (iii) availability of hospitalization status for the index episode of care. Exclusion criteria were: (i) duplicate entries; (ii) missing primary outcome data; and (iii) presentations clearly documented as traumatic chest pain. Because the study was based on retrospective registry data, the included population may still reflect some heterogeneity in chest pain etiology. Duplicate entries were identified using available registry identifiers, including patient identification code, date of presentation, and episode number when available. When more than one eligible entry was identified for the same patient during the study period, only the first eligible encounter was retained for analysis.

### 2.3. Data Collection and Variables

Data were extracted from hospital registries and medical records documented during the index emergency department encounter. Collected variables included demographics, comorbidities, clinical presentation, blood pressure, heart rate, troponin values, hospitalization status, and risk stratification scores. HEART and EDACS were analyzed as the main emergency department risk stratification scores. The Marburg Heart Score was also extracted when documented and analyzed as an exploratory complementary score, because it was originally developed mainly for primary care chest pain assessment rather than emergency department disposition decisions. The scores were not uniformly available for all patients; therefore, analyses involving risk scores were performed using available cases only. Troponin values were extracted as recorded in the hospital registry, were analyzed descriptively and were not used to define adjudicated myocardial infarction outcomes.

### 2.4. Outcome Measures

The primary outcome was hospitalization status during the index episode of care. Hospitalization was defined as admission from the emergency department to an inpatient hospital department or specialty unit, according to the registry entry. Patients discharged directly from the emergency department were classified as non-hospitalized. The original registry coding was recoded for analysis into two categories: hospitalized and non-hospitalized.

### 2.5. Statistical Analysis

Statistical analysis was performed using IBM SPSS Statistics version 23. Continuous variables were transformed into categorical variables to facilitate analysis. Categorical variables are presented as absolute frequencies and percentages. Continuous or ordinal variables are presented as medians and interquartile ranges when appropriate. For categorical analyses, contingency tables were constructed to examine associations between variables. Statistical significance was assessed using the chi-square test. Where applicable, degrees of freedom, *p*-values, and effect size estimates were reported. A *p*-value < 0.05 was considered statistically significant, while *p*-values < 0.001 were considered highly significant.

Continuous variables were categorized only when clinically relevant or required for predefined risk categories. Age was categorized into the following groups: 18–34, 35–54, 55–69, and ≥70 years. Risk scores were analyzed according to their established or predefined categories. The HEART score was categorized as low risk, intermediate risk, and high risk; EDACS was categorized as low risk and elevated risk; and the Marburg Heart Score was categorized as low risk and elevated risk.

Missing data were reported descriptively where present. Analyses were conducted using available-case analysis for each variable, and no imputation was performed. Patients with missing values for a specific variable were excluded only from analyses involving that variable.

Because of the retrospective registry-based design and the descriptive objective of the study, the analyses were primarily exploratory and based on unadjusted associations. No causal or independent predictive interpretation was inferred from chi-square analyses alone.

### 2.6. Ethical Consideration

This study followed the principles of the Helsinki Declaration on Medical Protocol and Ethics. Ethical approval was obtained from the Ethics Committee for Clinical Studies of Arad County Clinical Emergency Hospital (reference number 85 of 7 November 2024) and the Ethics Committee of the “Victor Babeş” University of Medicine and Pharmacy, Timişoara, Romania (reference number 86/01.11.2021 rev 2024). Patients provided general consent for data use upon hospital admission, as per standard clinical practice. Because this was a retrospective study based on anonymized registry and medical record data, no additional study-specific informed consent was required. All data were processed in an anonymized manner, and all procedures complied with institutional policies and applicable data protection regulations.

## 3. Results

### 3.1. Study Population

A total of 2070 adult patients presenting with acute chest pain were included in the final analysis after application of the eligibility criteria. The annual number of cases increased from 452 in 2021 to 563 in 2024. Overall, the largest proportions of patients were in the 35–54 and 55–69 age groups. Male patients predominated in the first three study years, while the sex distribution was nearly equal in 2024. Most patients were recorded as living in urban areas between 2021 and 2023, whereas a marked shift toward rural residence was observed in 2024. This finding should be interpreted cautiously, as it may reflect changes in registry coding, documentation practices, referral patterns, or catchment area characteristics rather than a definite epidemiological shift. Detailed year-by-year distributions are presented in Table 1. Overall four-year results are summarized in the text and in the “2021–2024” column of the tables, while detailed year-by-year data are presented for descriptive comparison.

### 3.2. Vascular Risk Factors and Comorbidities

Hypertension was the most prevalent comorbidity in all four study years, followed by diabetes mellitus. Previous myocardial infarction was present in a smaller but consistent proportion of patients, while neoplasms and chronic alcohol use were uncommon. Chronic smoking showed substantial variation across years, with the highest proportion recorded in 2024. Vascular risk factors and comorbidities by study year are presented in Table 2.

### 3.3. Clinical Presentation

Pressure-like chest pain was the predominant symptom across all study years, followed by stabbing and burning pain. Pain radiation, dyspnea, palpitations, exertional pain, and pain worsened by movement were less frequent but were consistently documented in the registry. Median blood pressure and heart rate at presentation were broadly stable across the study period. Detailed annual data on clinical presentation are presented in Table 3.

Also, cardiac troponin testing was available in most patients throughout the study period, including 424 (93.8%) patients in 2021, 503 (96.2%) in 2022, 503 (94.5%) in 2023, and 563 (100%) in 2024. Serial troponin measurements were documented in 30.0% of tested patients in 2021, 44.9% in 2022, 40.0% in 2023, and 49.0% in 2024. The median first recorded troponin value was 4.51 (1.19–16.35) in 2021, 8.13 (2.80–20.00) in 2022, 5.50 (0.62–15.50) in 2023, and 6.74 (1.20–25.00) in 2024. The corresponding median peak troponin values were 5.00 (1.25–20.00), 9.63 (3.18–23.80), 6.14 (1.15–16.95), and 9.00 (1.48–28.95), respectively. Detectable non-zero peak troponin values were observed in most tested patients, ranging from 91.1% in 2022 to 81.9% in 2024.

### 3.4. Risk Stratification Scores

The availability of risk score data differed slightly across study years. HEART score values were missing in 27 patients in 2021, 20 patients in 2022, and 31 patients in 2023, while no HEART score values were missing in 2024. EDACS values were available for all included patients. The Marburg Heart Score was available for all patients except one case in 2023.

Across all study years, the primary analysis focused on HEART and EDACS, as these are commonly used emergency department risk stratification tools for patients presenting with acute chest pain. Most patients were classified as low risk according to HEART and EDACS. Low-risk HEART scores accounted for the largest proportion of cases in most study years, while EDACS showed a stable distribution, with approximately two-thirds of patients classified as low risk. The Marburg Heart Score was retained only as an exploratory complementary score, because it was originally developed mainly for primary care chest pain assessment rather than emergency department disposition decisions. Detailed annual distributions of HEART, EDACS, and Marburg Heart Score categories are presented in Table 4.

Most patients were classified as low risk according to HEART in 2021–2023, whereas in 2024, a higher proportion of intermediate-risk cases was observed. EDACS categories remained relatively stable across all study years, with approximately two-thirds of patients classified as low risk. For the Marburg Heart Score, most patients fell into the elevated-risk category throughout the study period.

### 3.5. Hospitalization Status

Hospitalization status was analyzed in relation to the available risk stratification scores. In unadjusted analyses, HEART score categories were significantly associated with hospitalization status across all study years: 2021 (χ^2^ = 88.76, *p* < 0.001), 2022 (χ^2^ = 53.06, *p* < 0.001), 2023 (χ^2^ = 38.40, *p* < 0.001), and 2024 (χ^2^ = 140.52, *p* < 0.001). EDACS categories were also significantly associated with hospitalization status in 2021 (χ^2^ = 42.73, *p* < 0.001), 2022 (χ^2^ = 38.98, *p* < 0.001), 2023 (χ^2^ = 32.34, *p* < 0.001), and 2024 (χ^2^ = 63.26, *p* < 0.001). Similar statistically significant associations were observed for the Marburg Heart Score in 2021 (χ^2^ = 67.57, *p* < 0.001), 2022 (χ^2^ = 46.87, *p* < 0.001), 2023 (χ^2^ = 37.10, *p* < 0.001), and 2024 (χ^2^ = 68.74, *p* < 0.001).

Descriptively, hospitalized patients had higher median HEART and EDACS values than non-hospitalized patients across all study years. In 2021, the hospitalized patients showed a median HEART score of 4 (3–5) compared with 3 (2–4) in the non-hospitalized group. The corresponding median EDACS values were 16 (12–21) and 10 (7–14), respectively. A similar pattern was observed in the 2022 subset, where hospitalized patients had higher median HEART (4 versus 3) and EDACS (16 versus 11) scores. In 2023, the same gradient persisted, with median HEART values of 4 (3–4) versus 3 (2–4) and median EDACS values of 16 (12–20) versus 11 (7–16) in the hospitalized and non-hospitalized groups, respectively. The separation was most pronounced in 2024, when the median HEART score reached 5 in the hospitalized group compared with 3 among non-hospitalized patients, while median EDACS values were 17 and 8, respectively. The Marburg Heart Score also tended to be higher among hospitalized patients, although the difference was less marked than for HEART and EDACS. Overall, these findings suggest that higher HEART and EDACS values were associated with a greater likelihood of hospitalization, with the clearest distinction observed in 2024.

These findings indicate that hospitalization status was associated with higher risk score categories in unadjusted analyses, particularly for HEART and the Marburg Heart Score. These results support the descriptive role of structured risk assessment in the initial evaluation of emergency department patients presenting with acute chest pain, while not implying independent prediction in the absence of multivariable adjustment.

## 4. Discussion

### 4.1. Associations Between Study Variables

The present study evaluated the clinical profile, cardiovascular risk burden, and risk stratification patterns of patients presenting with acute chest pain over a four-year period.

First, in unadjusted analyses, structured risk stratification scores were significantly associated with hospitalization status across the study years. Second, age emerged as the sociodemographic factor most consistently associated with cardiovascular comorbidity burden, being repeatedly associated with hypertension, diabetes mellitus, previous myocardial infarction, and, in most years, dyslipidemia. Third, age was also strongly associated with both HEART and Marburg score categories throughout the study period, whereas sex and area of residence showed fewer and less consistent associations. Taken together, these findings suggest that, in this cohort, acute chest pain assessment was shaped primarily by baseline cardiovascular risk and age-related clinical complexity rather than by broader sociodemographic characteristics.

One of the most clinically relevant observations was the consistent association between structured risk stratification scores and hospitalization status. In unadjusted analyses, HEART, EDACS, and Marburg Heart Score categories were significantly associated with hospitalization status across the study years. These findings suggest that patients classified into higher risk categories were more frequently hospitalized. However, because the present analysis was based on contingency tables and chi-square testing, these results should be interpreted as unadjusted associations rather than evidence of independent prediction. These findings suggest an association between higher-risk score categories and hospitalization status in patients presenting with acute chest pain.

Age was the factor most consistently associated with comorbidity burden in the cohort. Significant associations between age and hypertension were present in all four study years, with highly significant *p*-values throughout. A similar pattern was observed for diabetes and previous myocardial infarction, both of which were significantly associated with age in every year analyzed. Dyslipidemia also showed significant age-related variation in 2021, 2023, and 2024, although this association did not reach statistical significance in 2022. Clinically, these results are plausible and reinforce the well-established observation that older patients presenting with chest pain tend to accumulate a greater burden of traditional cardiovascular risk factors and prior cardiovascular disease. In practical terms, this means that age in this cohort was not merely a demographic descriptor, but a major marker of baseline cardiovascular vulnerability.

A strong association was also observed between age and risk score categories. This finding should be interpreted cautiously because age is itself a component of several risk stratification tools, including HEART and EDACS. Therefore, the observed relationship between age and higher score categories partly reflects the structure of the scores rather than an independent clinical association. For this reason, age-related findings were interpreted descriptively and were not considered evidence of independent predictive value.

By contrast, sex and area of residence had a more limited and inconsistent relationship with both comorbidities and risk score categories. Sex was not consistently associated with hypertension, diabetes, or dyslipidemia across the study years, and residence status showed no significant association with these major cardiovascular comorbidities. Likewise, sex was significantly associated with HEART only in 2024, and with Marburg only in 2021 and 2024, while rural versus urban residence showed a significant relationship with HEART only in 2024 and no significant association with Marburg. These findings suggest that, within this cohort, age was more consistently associated with cardiovascular risk burden and risk score distribution. Isolated year-specific associations may reflect differences in cohort composition, documentation practices, or local referral patterns rather than reproducible biological or epidemiological effects.

Another relevant aspect of the present study is the integration of cardiac troponin assessment into the initial evaluation of chest pain. Troponin testing was available in most patients across the study period, suggesting that biomarker-based assessment was part of routine emergency department practice in the evaluated setting. However, troponin-related findings should be interpreted cautiously. Assay type, analytical platform, measurement units, laboratory-specific cut-off values, and timing of serial measurements were not consistently available in the retrospective registry. Therefore, troponin values were analyzed descriptively and were not used to define adjudicated myocardial infarction outcomes.

From a broader clinical perspective, the findings of this study suggest that the evaluation of acute chest pain in everyday practice benefits from combining structured scores with the patient’s underlying cardiovascular profile. The repeated association between higher-risk score categories and hospitalization, together with the strong contribution of age and comorbidity burden, suggests that structured risk assessment may contribute to the overall clinical evaluation of patients presenting with acute chest pain. In this cohort, the most informative pattern was not a demographic distinction between men and women or between rural and urban patients, but the clustering of cardiovascular comorbidity and higher-risk score categories in older patients. This observation may help explain why older patients with acute chest pain often require more intensive diagnostic evaluation and closer clinical monitoring.

In summary, this four-year retrospective analysis showed that HEART, EDACS, and Marburg Heart Score categories were associated with hospitalization status in unadjusted analyses. These findings reflect real-world disposition patterns in a single emergency department and should not be interpreted as validation of independent prognostic performance.

### 4.2. Comparison with Existing Literature

This section places the findings of the present study in the context of the existing literature on the assessment of acute chest pain and cardiovascular risk stratification.

Risk stratification tools have been extensively investigated in patients presenting with chest pain, particularly in emergency department settings. The HEART score is one of the most widely validated instruments for the early assessment of patients with acute chest pain, integrating history, electrocardiographic findings, age, cardiovascular risk factors, and troponin values [9]. Previous validation studies have shown that low HEART scores identify patients with a low short-term risk of major adverse cardiac events, supporting its use as part of structured emergency department assessment pathways [10,11]. EDACS was developed as part of an accelerated diagnostic protocol to identify patients with chest pain who may be suitable for early discharge after short-term evaluation [12,13,14]. In the present study, most patients were classified as low risk according to EDACS, and EDACS categories were significantly associated with hospitalization status in unadjusted analyses. However, because hospitalization was used as the outcome rather than adjudicated major adverse cardiac events, direct comparison with EDACS validation studies should be made cautiously.

The Marburg Heart Score was originally developed and validated mainly for primary care patients with chest pain, with the purpose of helping clinicians rule out coronary heart disease [15]. Although its clinical context differs from that of emergency department risk stratification tools such as HEART and EDACS, its inclusion in the present study provides an additional perspective on symptom-based and risk-factor-based assessment. The finding that Marburg Heart Score categories were associated with hospitalization status should therefore be interpreted descriptively and in relation to local clinical decision-making, rather than as validation of the score for emergency department disposition decisions.

Previous studies have described acute chest pain as a major challenge in emergency medicine, emphasizing the importance of early assessment and appropriate triage for identifying high-risk patients, as well as the role of ECG and cardiac troponin testing in the initial evaluation of patients presenting to the emergency department [16]. In elderly patients with cardiovascular disease, age has been identified as an independent factor associated with mortality, suggesting that advancing age influences both the manifestation of symptoms and clinical progression [17]. In line with these observations, age is also a major determining factor in the etiology of acute chest pain, with older patients having a significantly higher probability of cardiac causes compared to younger individuals. For example, Khan et al. reported that patients over the age of 60 have a significantly higher probability of cardiac etiologies, highlighting the link between age and cardiovascular risk [18]. Similarly, other studies have shown that advancing age significantly influences the cardiovascular profile of patients, being associated with a higher prevalence of cardiovascular conditions and associated risk factors among older age groups. These factors, which include diabetes, high blood pressure, and a family history of cardiovascular disease, further confirm the central role of age in determining overall cardiovascular risk [19]. Studies have also suggested that age may influence the clinical presentation of chest pain. For example, older patients with acute myocardial infarction have been found to experience less severe pain compared to younger adults, indicating that symptom perception and reporting may differ depending on age [20]. In addition, Grosmaitre et al. reported that older patients are less likely to experience typical chest pain and more likely to present with atypical symptoms, which can lead to delayed hospital presentation, a more severe condition at hospitalization, and less favorable short-term clinical outcomes [21]. These findings are consistent with the present study, in which age emerged as the sociodemographic factor most consistently associated with cardiovascular comorbidity burden.

According to our results, comorbidity burden was an important factor influencing cardiovascular risk in patients presenting with acute chest pain. It has been reported that patients presenting with acute chest pain often have multiple comorbidities, such as diabetes, a history of cardiovascular disease, and chronic lung conditions, which can influence their clinical evolution as well as the time they spend in the hospital [22]. The literature also suggests that chest pain is associated with an increased risk of adverse cardiovascular events, including myocardial infarction and heart failure, even after adjusting for standard risk factors [23].

Overall, these findings highlight the multifactorial nature of cardiovascular risk in patients with acute chest pain, where age and comorbidities play a central role in both risk stratification and clinical outcomes.

## 5. Limitations

The limited availability of detailed data on final diagnosis, treatment administered, evolution after discharge, or mortality has limited the evaluation of more specific clinical outcomes. First, the retrospective single-center registry-based design limits causal inference and may affect the generalizability of the findings. Data completeness depended on the quality of routine clinical documentation. Second, although acute chest pain was defined according to the documented presenting complaint, the study population may have included heterogeneous cardiac and non-cardiac etiologies. Final diagnostic categories, treatment data, length of hospitalization, readmission, mortality, and short-term adverse cardiac events were not consistently available in a standardized format. Third, hospitalization status was used as the primary outcome. This pragmatic disposition outcome may be influenced by local protocols, bed availability, physician judgment, emergency department workflow, social factors, and referral pathways, and should not be interpreted as a direct surrogate for disease severity or adverse cardiovascular prognosis.

Fourth, HEART, EDACS, and Marburg Heart Score values were extracted as documented in the registry or medical records and were not retrospectively recalculated. Score documentation was not uniform across all patients and study years; therefore, analyses involving risk scores were performed using available cases only, without imputation. Troponin values were also analyzed descriptively, as assay type, analytical platform, measurement units, laboratory-specific cut-off values, and upper reference limits were not consistently retrievable. The marked change in residence distribution observed in 2024 should also be interpreted cautiously, as it may reflect changes in registry coding, documentation practices, referral patterns, or extraction procedures rather than a definite epidemiological shift.

Finally, the statistical analysis was primarily descriptive and based on unadjusted associations. No multivariable logistic regression or discrimination analyses, such as ROC/AUC, sensitivity, specificity, or negative predictive value, were performed. Therefore, the present study cannot assess the independent predictive performance of HEART, EDACS, or the Marburg Heart Score. Future prospective multicenter studies using standardized diagnostic outcomes and follow-up data are needed.

## 6. Conclusions

This single-center retrospective four-year study of emergency department patients presenting with acute chest pain described clinical characteristics, cardiovascular comorbidity burden, risk score distribution, and hospitalization status. HEART, EDACS, and Marburg Heart Score categories were associated with hospitalization status in unadjusted analyses, while age was associated with cardiovascular comorbidity burden and risk score categories. However, these findings should be interpreted as descriptive registry-based associations rather than evidence of independent predictive performance. The results highlight the importance of integrated clinical assessment in acute chest pain, while underscoring the need for future studies using standardized diagnostic outcomes, follow-up data, and multivariable analyses.

## Figures and Tables

**Table 1:** Baseline characteristics of the study population by study year.

Characteristics/Year	2021	2022	2023	2024	2021–2024
**Age group, N (%)**					
18–34 years old	61 (13)	64 (12)	82 (15)	78 (14)	285 (13.8)
35–54 years old	164 (36)	188 (36)	169 (32)	189 (33)	710 (34.3)
55–69 years old	129 (29)	152 (29)	141 (27)	168 (30)	590 (28.5)
≥ 70 years old	98 (22)	119 (23)	140 (26)	128 (23)	485 (23.4)
**Sex, N (%)**					
Female	194 (43)	243 (46)	246 (46)	279 (50)	962 (46.5)
Male	258 (57)	280 (54)	286 (54)	284 (50)	1108 (53.5)
**Area of Residence, N (%)**					
Rural	176 (39)	248 (47)	208 (39)	514 (91)	1146 (55.4)
Urban	276 (61)	275 (53)	324 (61)	49 (9)	924 (44.6)

**Table 2:** Baseline characteristics of the vascular risk factors and comorbidities by study year.

Vascular Risk Factors and Comorbidities/Year	2021	2022	2023	2024	2021–2024
	N (%)	N (%)	N (%)	N (%)	
Hypertension	217 (48.0)	267 (51.1)	268 (50.4)	289 (51.3)	1041 (50.3)
Diabetes	116 (25.7)	158 (30.2)	182 (34.2)	188 (33.4)	644 (31.1)
Dyslipidemia	95 (21.0)	52 (9.9)	66 (12.4)	83 (14.7)	296 (14.3)
Previous myocardial infarction	62 (13.7)	70 (13.4)	80 (15.0)	64 (11.4)	276 (13.3)
Neoplasms	6 (1.3)	4 (0.8)	10 (1.9)	11 (2.0)	31 (1.5)
Chronic smoking	41 (9.1)	59 (11.3)	52 (9.8)	133 (23.6)	285 (13.8)
Chronic alcohol use	14 (3.1)	5 (1.0)	13 (2.4)	23 (4.1)	55 (2.7)

**Table 3:** Clinical presentation according to study year.

Characteristics/Year	2021 (N = 452, %)	2022 (N = 523, %)	2023 (N = 532, %)	2024 (N = 563, %)	2021–2024
Pressure-like pain	353 (78.1)	393 (75.1)	339 (63.7)	382 (67.9)	1467 (70.9)
Stabbing pain	60 (13.3)	103 (19.7)	158 (29.7)	127 (22.6)	448 (21.6)
Burning pain	32 (7.1)	25 (4.8)	32 (6.0)	53 (9.4)	142 (6.9)
Other pain descriptors	7 (1.5)	2 (0.4)	3 (0.6)	1 (0.2)	13 (0.6)
Pain radiation	70 (15.5)	126 (24.1)	109 (20.5)	122 (21.7)	427 (20.6)
Dyspnea	28 (6.2)	57 (10.9)	58 (10.9)	63 (11.2)	206 (10.0)
Palpitations	27 (6.0)	59 (11.3)	57 (10.7)	43 (7.6)	186 (9.0)
Exertional pain	17 (3.8)	38 (7.3)	69 (13.0)	61 (10.8)	185 (8.9)
Pain worsened by movement	29 (6.4)	64 (12.2)	83 (15.6)	52 (9.2)	228 (11.0)
Median BP, mmHg	140/80	140/80	140/80	140/80	-
Median HR, bpm	80.5	80	80	80	-

**Table 4:** Distribution of risk stratification scores by study year.

Risk Stratification Scores	2021 (N = 452)	2022 (N = 523)	2023 (N = 532)	2024 (N = 563)	2021–2024
**Main emergency department scores—HEART Score, N (%)**					
Low risk (0–3)	246 (54.1)	271 (51.8)	301 (56.6)	265 (47.1)	1083 (52.3)
Intermediate risk (4–6)	168 (37.2)	218 (41.7)	198 (37.2)	262 (46.5)	846 (40.9)
High risk (7–10)	11 (2.4)	14 (2.7)	2 (0.4)	36 (6.4)	63 (3.0)
Missing medical record	27 (6.0)	20 (3.8)	31 (5.8)	0	78 (3.8)
**Main emergency department scores—EDACS Score, N (%)**					
Low risk (<16)	302 (66.8)	335 (64.1)	354 (66.5)	378 (67.1)	1369 (66.1)
Elevated risk (≥16)	150 (33.2)	188 (35.9)	178 (33.5)	185 (32.9)	701 (33.9)
**Exploratory complementary score—Marburg Heart Score, N (%)**					
Low risk (0–2)	165 (36.5)	152 (29.1)	225 (42.3)	184 (32.7)	726 (35.1)
Elevated risk (=>3)	287 (63.5)	371 (70.9)	306 (57.5)	379 (67.3)	1343 (64.9)
Missing medical record	0	0	1 (0.2)	0	1 (0.05)

## Data Availability

The original contributions presented in this study are included in the article. Further inquiries can be directed to the corresponding author.
